# Systematic evaluation of *C. elegans* lincRNAs with CRISPR knockout mutants

**DOI:** 10.1186/s13059-018-1619-6

**Published:** 2019-01-08

**Authors:** Shuai Wei, He Chen, Emmanuel Enoch Dzakah, Bin Yu, Xiaolin Wang, Tao Fu, Jingxin Li, Lei Liu, Shucheng Fang, Weihong Liu, Ge Shan

**Affiliations:** 1 Division of Molecular Medicine, Hefei National Laboratory for Physical Sciences at Microscale, the CAS Key Laboratory of Innate Immunity and Chronic Disease, School of Life Sciences, University of Science and Technology of China, Hefei, 230027 China; 2 Department of Molecular Biology and Biotechnology, School of Biological Sciences, College of Agriculture and Natural Sciences, University of Cape Coast, Cape Coast, Ghana; 3 MOE Key Laboratory of Bioinformatics, Center for Synthetic and Systems Biology, School of Life Sciences, Tsinghua University, Beijing, 100084 China; 4 Present address: Department of Molecular, Cellular, and Developmental Biology, University of Colorado, Boulder, CO 80309 USA; 5 Present address: Hanwang Technology Co., Ltd., Haidian District, Beijing, 100193 China; 6 CAS Center for Excellence in Molecular Cell Science, Shanghai Institute of Biochemistry and Cell Biology, CAS, Shanghai, 200031 China

**Keywords:** *C. elegans*, lincRNA, MicroRNA, CRISPR, Phenotype, Transcription factor

## Abstract

**Background:**

Long intergenic RNAs (lincRNAs) play critical roles in eukaryotic cells, but systematic analyses of the lincRNAs of an animal for phenotypes are lacking. We generate CRISPR knockout strains for *Caenorhabditis elegans* lincRNAs and evaluate their phenotypes.

**Results:**

*C. elegans* lincRNAs demonstrate global features such as shorter length and fewer exons than mRNAs. For the systematic evaluation of *C. elegans* lincRNAs, we produce CRISPR knockout strains for 155 of the total 170 *C. elegans* lincRNAs. Mutants of 23 lincRNAs show phenotypes in 6 analyzed traits. We investigate these lincRNAs by phenotype for their gene expression patterns and potential functional mechanisms. Some *C. elegans* lincRNAs play cis roles to modulate the expression of their neighboring genes, and several lincRNAs play trans roles as ceRNAs against microRNAs. We also examine the regulation of lincRNA expression by transcription factors, and we dissect the pathway by which two transcription factors, UNC-30 and UNC-55, together control the expression of *linc-73*. Furthermore, linc-73 possesses a *cis* function to modulate the expression of its neighboring kinesin gene *unc-104* and thus plays roles in *C. elegans* locomotion.

**Conclusions:**

By using CRISPR/cas9 technology, we generate knockout strains of 155 *C. elegans* lincRNAs as valuable resources for studies in noncoding RNAs, and we provide biological insights for 23 lincRNAs with the phenotypes identified in this study.

**Electronic supplementary material:**

The online version of this article (10.1186/s13059-018-1619-6) contains supplementary material, which is available to authorized users.

## Background

Long intergenic RNAs (lincRNAs) are a specific class of long noncoding RNAs (lncRNAs) that are encoded by genomic sequences without overlap with genomic sequences of known coding genes [1, 2]. LincRNAs were identified first in mammalian cells, and they are key regulators of diverse biological processes such as transcription and chromatin epigenetics [3, 4]. Mutations in lincRNAs have been shown to promote the development of many complex diseases, such as inflammation, viral infection, and carcinogenesis [3, 5, 6]. For example, one extensively studied lincRNA, hotair, regulates epidermal differentiation and associates with cancer metastasis by interacting with epigenetic factors such as Polycomb repressive complex 2 (PRC2) [7, 8]. LincRNA-p21 has been shown to play crucial roles in hypoxia-enhanced glycolysis by forming a positive feedback loop between HIF-1α and lincRNA-p21 to enhance glycolysis under hypoxia [9]. These roles have been characterized mostly with cultured cells, tumor xerographs, tissues, and only recently and for a very limited number of lincRNAs, also at the whole organismal level [10, 11]. For example, linc1405 has recently been found to modulate the Eomes/WDR5/GCN5 complex in mouse ESCs, and at the whole animal level, depletion of linc1405 impedes heart development in mice [10]. In another study, lincRNA-EPS was found to play a *trans* role in recruiting the heterochromatin binding protein hnRNP L to control nucleosome positioning and inhibit the transcription of immune response genes, and lincRNA-EPS traditional knockout mice demonstrate enhanced inflammation [11].

Hundreds of lincRNAs have also been identified in other metazoans such as *Caenorhabditis elegans*, *Drosophila*, and zebrafish [12–14]. There are 170 lincRNAs encoded in the current annotated *C. elegans* genome [15, 16]. Thus far, little is known about the functions and phenotypes associated with these *C. elegans* lincRNAs. Furthermore, there has been essentially no systematic analysis of all lincRNAs with knockout strains for any given animal.

CRISPR technology enables efficient production of *C. elegans* knockout and insertion strains [17–23]. In this study, we generated knockout strains using CRISPR for 155 of the 170 *C. elegans* lincRNAs. Among the 6 traits we analyzed, mutants of 23 lincRNAs exhibited phenotypes. We also provided mechanistic insights for these lincRNAs.

## Results

### Genome-wide characteristics of *C. elegans* lincRNAs

**Fig. 1:**
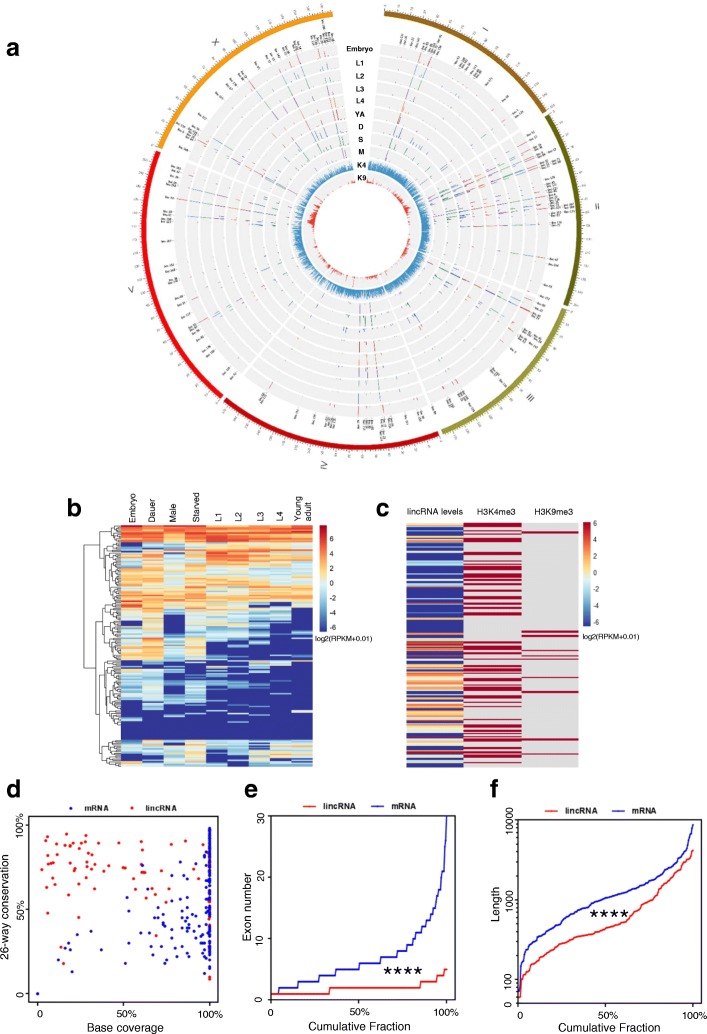
Genomic characterization of *C. elegans* lincRNAs. **a** Circos plot of the 170 lincRNAs in the *C. elegans* genome. The expression levels of 170 lincRNAs in nine developmental stages and populations: embryo, L1, L2, L3, and L4, YA (young adult), D (dauer), S (mixed stages of worms under starvation), and M (male, him-5 mix worms) are shown in the inner tracks. The two innermost tracks represent distributions of H3K4me3 (K4) and H3K9me3 (K9) ChIP-seq signals (L4 worms), at the whole genome (not just for lincRNA genes). **b** Hierarchical clustering of the relative expression levels of the 170 lincRNAs. RNA-seq data from 9 developmental stages were normalized to log2 (RPKM+ 0.01). **c** lincRNA expression levels (heatmap of RPKM) along with H3K4me3 and H3K9me3 binding (binary map with binding in red) on lincRNA genes. **d** Conservation score of lincRNAs and mRNAs (*n* = 200, randomly chosen). “Base coverage” refers to the percentage of annotated bases. Conservation phastCons scores of 26 nematodes were interrogated from the UCSC genome browser [61], and the degree of conservation along with the portion of conserved sequences to the full-length (base coverage) lincRNAs and mRNAs were compared. **e** Cumulative plot of exon numbers of randomly selected lincRNAs and mRNAs (*n* = 200, randomly chosen). **f** Length distribution of lincRNAs and mRNAs (*n* = 200, randomly chosen). For the analysis of sequence conservation, 26 nematode conservation phastCons scores were interrogated from UCSC [61] for each base of an individual *C. elegans* lincRNA or mRNA, and the scores of each transcript were averaged. For d and e, ****, *p* < 0.0001 by the two-sided Mann-Whitney *U* test

We performed H3K4me3 and H3K9me3 ChIP-seq and sequenced the expression profiles of embryos, L1 stage, L2 stage, dauer stage, L3 stage, L4 stage, young adults, males (*him-5* worms), and mixed stages of worms under starvation and then analyzed the 170 *C. elegans* lincRNAs for their global features (Fig. 1a, b). Several lincRNAs showed stage-specific expression (Fig. 1b, Additional file 1: Table S1). For example, linc-28, linc-131, and linc-155 were expressed only in embryos; linc-148 was expressed exclusively in L2 worms; linc-52 was expressed in young adults only; linc-141 and linc-168 were expressed only in dauer; and linc-23 was expressed in males only (Additional file 1: Table S1). There were 12 lincRNAs expressed at all stages examined, and their expression levels showed low variations (the ratio of the highest to the lowest levels of each lincRNA respectively was within tenfold) (Additional file 1: Table S1). These results indicated that the expression of some lincRNAs was under tight control for stage-specific expression and functions, while some other lincRNAs might play ubiquitous roles with expression at all stages. H3K4me3 is generally an activation marker, and H3K9me3 is a suppressive marker. We noticed that in L4 worms, H3K4me3 bound to genomic regions of the majority of lincRNAs, although H3K9me3 only bound to genomic regions of 12 lincRNAs (Fig. 1c). These results suggested a dynamic and regulated expression of *C. elegans* lincRNAs, and further investigations are necessary to dissect the relevant mechanisms and factors such as transcription factors and histone modifications.

Compared to mRNAs, lincRNAs were less conserved in 26 nematode species (Fig. 1c). When there were conserved sequences, the length of these sequences was also shorter in lincRNAs than in mRNAs (Fig. 1c). The exon numbers of lincRNAs were significantly fewer than of mRNAs (Fig. 1d). lincRNAs were also significantly shorter than mRNAs (Fig. 1e). These features of exon numbers and sequence length were also true for lincRNAs in several other organisms [1, 12].

### Phenotypes of lincRNA CRISPR knockout strains

**Fig. 2:**
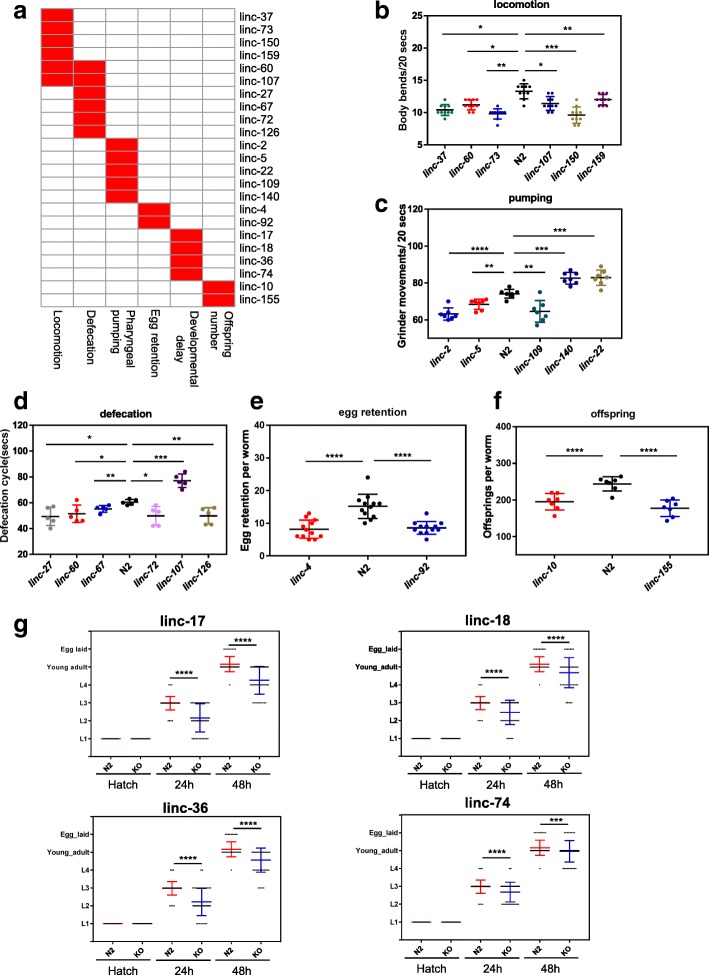
Phenotypic analysis of lincRNA mutants. **a** Summary of the phenotypic characteristics of lincRNA mutants. Six phenotypic traits (locomotion, defecation, pharyngeal pumping, egg retention, rate of development, and number of progeny) were examined in 155 lincRNA mutants. The red cell represents phenotypic data of the corresponding lincRNA mutant that were with statistically significantly different compared with the wild-type data. **b** Six lincRNA mutants showed uncoordination. **c** Six lincRNA mutants had defects in defecation. **d** Five lincRNA mutants showed defects in pharyngeal pumping. **e** Two lincRNA mutants showed egg retention defects. **f** Two lincRNA mutants had deceased numbers of progeny. **g** Four lincRNA mutants demonstrated a delay in development. *n* = 50. For g, data for N2 worms were reused in the figure for comparison to lincRNA mutants. For **b**–**f**, *p < 0.05; **, *p* < 0.01; *** *p* < 0.001; **** *p* < 0.0001; *p* values were calculated by the unpaired Student’s *t* test; for **g**, ***, p < 0.001, **** *p* < 0.0001, *p* values were calculated by the chi-square test

To investigate the roles of these lincRNAs, we generated CRISPR knockout (KO) strains of 155 *C. elegans* lincRNAs (Additional file 2: Figure S1, Additional file 3: Table S2). None of the 155 lincRNA mutants showed obvious abnormality in morphology, and they did not have a severe lethal phenotype. Actually, for the 15 lincRNAs we failed to obtain CRISPR knockouts, the failure might be technical and was not due to lethality of mutants as we did not even get heterozygotes. We then examined the locomotion, defecation, pharyngeal pumping, egg retention, developmental delay, and offspring numbers of these KO strains. Twenty-three lincRNA KO strains showed defects in these 6 traits (Fig. 2, Additional file 4: Table S3); 6 lincRNAs (linc-37, linc-60, linc-73, linc-107, linc-150, and linc-159) showed uncoordination (Fig. 2a, b); 6 lincRNAs (linc-27, linc-60, linc-67, linc-72, linc-107, and linc-126) had defects in defecation (Fig. 2a, c); 5 lincRNAs (linc-2, linc-5, linc-22, linc-109, and linc-140) showed defects in pharyngeal pumping (Fig. 2a, d); 2 lincRNAs (linc-4 and linc-92) showed egg retention (Fig. 2a, e); and 2 lincRNAs, linc-10 and linc-155 had deceased numbers of progeny (Fig. 2a, f). linc-10 and linc-155 mutants actually laid fewer eggs, although essentially all eggs hatched. Four lincRNAs (linc-17, linc-18, linc-36, and linc-74) demonstrated a delay in development (Fig. 2a, g). Two lincRNAs, linc-60 and linc-107, showed pleiotropic effects in locomotion and defecation (Fig. 2a, b, d).

### Expression patterns of lincRNAs with a mutant phenotype

**Fig. 3:**
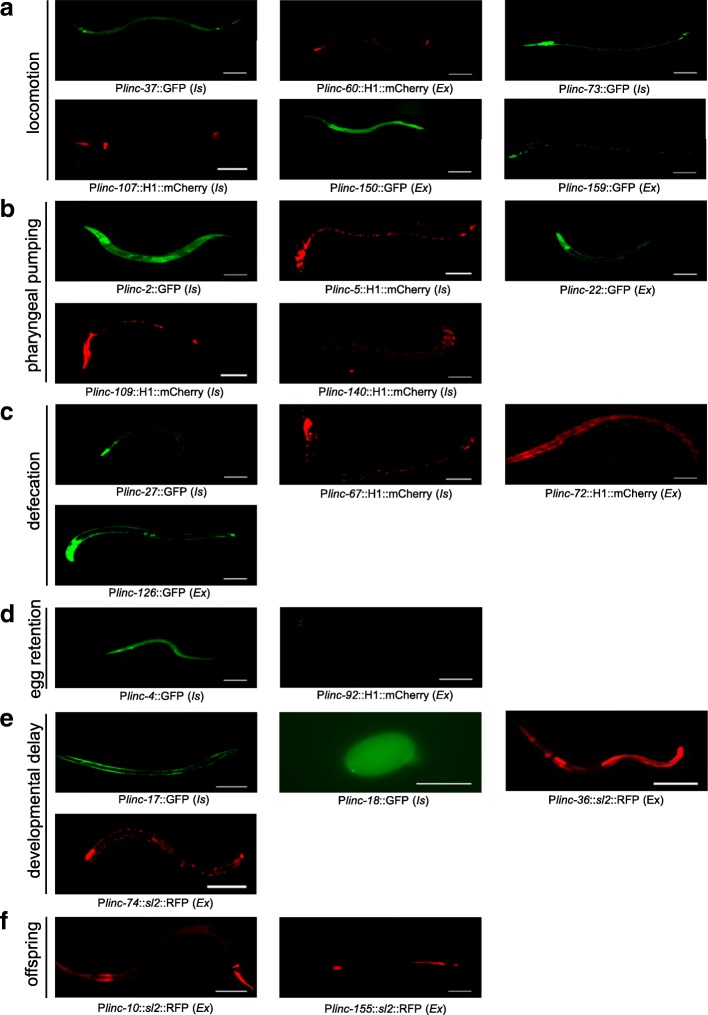
Transcriptional reporters of lincRNAs. **a** The expression of transcriptional reporters of lincRNAs with locomotion defects. **b** The expression of transcriptional reporters of lincRNAs with defects in pharyngeal pumping. **c** The expression of transcriptional reporters of lincRNAs with defecation defects. **d** The expression of transcriptional reporters of lincRNAs with defects in egg retention. **e** The expression of transcriptional reporters of lincRNAs with developmental delay. **f** The expression of transcriptional reporters of lincRNAs with a decreased number of progeny. Is, integrated strain; Ex, extrachromosomal strain. Scale bar, 50 μm

Next, we examined the expression of lincRNAs with phenotypes using transcriptional reporter (Fig. 3). For the six lincRNAs with the uncoordination phenotype, we noticed that five (excluding linc-107) were expressed in neurons and/or muscles (Fig. 3a). For the five lincRNAs with defects in pharyngeal pumping, four (excluding linc-140) showed expression in pharyngeal muscles and neurons (Fig. 3b). The expression patterns indicated that these nine lincRNAs were expressed in cells in association with their specific phenotypes, and thus, they might play cell-autonomous roles. The other four phenotypes, defecation, egg retention, developmental delay, and offspring numbers, were relatively more complex and might be related to multiple cell types; thus, a direct link between the expression patterns of the lincRNA and the corresponding phenotype was difficult to establish (Fig. 3d–f). Additionally, 14 lincRNAs (*Is* strains) had integrated reporters and 9 lincRNAs (*Ex* strains) had non-integrated extrachromosomal reporters (Fig. 3, Additional file 3: Table S2).

### Correlations between lincRNAs and mRNAs

**Fig. 4:**
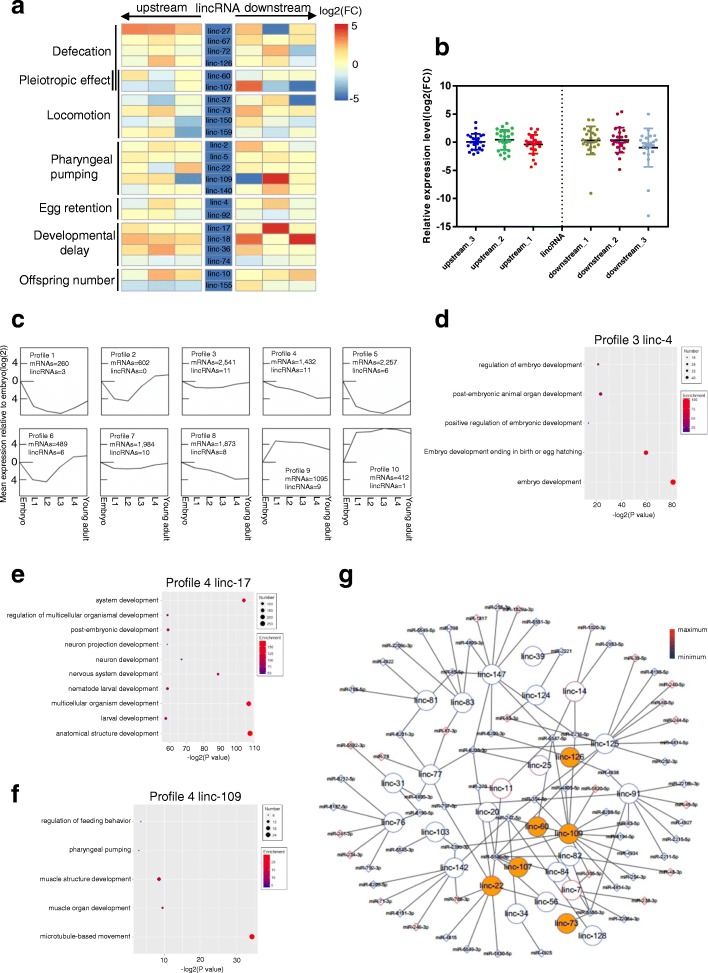
Connections of lincRNAs to mRNAs and microRNAs. **a** Heatmap of the expression levels of lincRNA-neighboring genes in lincRNA mutants. The expression level of each gene was assessed by qRT-PCR, and log_2_(FC) compared to wild type in gene expression was converted to heatmap (FC, fold change). Forward and backward arrows indicate the downstream and upstream genes. Log_2_(FC) were set between − 5 and 5 mandatorily to draw the heat map. **b** Relative expression levels of neighboring genes of the 23 lincRNAs with mutant phenotypes; data are the same as in a, except that the Log_2_(FC) are actual values. **c** Mean expression profiles of mRNAs and lincRNAs using our RNA-seq data from six developmental stages (embryo, L1, L2, L3, L4, young adult). Data were analyzed by Short Time-series Expression Miner (STEM) [24] using k-means clustering. Signals for each profile cluster were normalized to signals of the embryonic stage. **d** GO analysis of coding genes in profile 3 for ontology matching the *linc-4* phenotype of egg retention. **e** GO analysis of protein coding genes in profile 4 for ontology matching the *linc-17* phenotype of developmental delay. **f** GO analysis of protein-coding genes in profile 4 for ontology matching the *linc-109* phenotype of pharyngeal pumping. **g** Global network of the lincRNA-miRNA interaction constructed with our RNA-seq data for long RNAs and microRNAs from nine developmental stages and worm populations. LincRNAs in golden brown-filled circles represent lincRNAs with mutant phenotypes in this study. The line colors of the circle (for lincRNA) and diamond (for miRNA) represent relative expression levels (scale shown to the right)

For the lincRNAs with a mutant phenotype, we examined whether they affected the expression of their neighboring genes (Fig. 4a, b). For certain lincRNAs such as linc-67, linc-5, and linc-74, there were no substantial changes in the expression levels of their adjacent genes once the lincRNAs were knocked out (Fig. 4a). For lincRNAs such as linc-17 and linc-18, there were significant increases in the expression levels of their adjacent genes in the corresponding knockouts (Fig. 4a). Interestingly, the majority of these 23 lincRNAs showed complex effects on the expression of neighboring genes, with some adjacent genes demonstrating increased expression levels and some other adjacent genes simultaneously exhibiting decreased expression levels in the knockouts (Fig. 4a). When considered as a whole, the positions of neighboring genes from the lincRNA locus showed no specific trend in how lincRNAs affected their neighboring genes (Fig. 4b). These results indicated that some of these lincRNAs had *cis* effects on the expression of their neighboring genes, and they could either activate and/or suppress gene expression. For each individual lincRNA, however, further experiments are necessary to validate the potential *cis* role.

We also analyzed the expression correlations between the lincRNAs and the corresponding coding genes within the 100 kb upstream and downstream genomic regions (Additional file 5: Figure S2a, b); for either all the 170 lincRNAs or the 23 lincRNAs with phenotypes, the correlation between the expression of lincRNAs and mRNAs seemed to have no relevance to the position of the mRNA from the lincRNA locus. We further examined the relationship between the mean expression profiles of mRNAs and lincRNAs based on RNA-seq data for embryos, L1, L2, L3, and L4, and young adults generated by our group using Short Time-series Expression Miner (STEM) [24]. Ten expression profile patterns were obtained after normalizing the mean expression of both lincRNAs and mRNAs in L1, L2, L3, and L4, and young adults to the mean expression in the embryo (Fig. 4c). Nine of the 10 expression profiles (missing the expression profile pattern 2) contained lincRNAs that showed a correlated expression similar to the mRNAs. In these 10 expression profile patterns, profile patterns 3 and 4 showed an enrichment for the largest number of lincRNAs (11 lincRNAs in each pattern) (Fig. 4c). Gene ontology (GO) analysis of coding genes in profile 3 revealed enrichment for genes involved in the regulation of embryonic development and embryo development ending in birth or egg hatching, among others (Fig. 4d). Among the 11 lincRNAs in profile 3, only one lincRNA, linc-4, had a phenotype (egg retention) (Figs. 2a and 4d). Among the 11 lincRNAs in profile 4, two lincRNAs, linc-17 (developmental delay) and linc-109 (pharyngeal pumping), had phenotypes (Fig. 2a). GO terms in profile 4 showed enrichment for genes in system development, larval development, and pharyngeal pumping (Fig. 4e, f).

### Interactions between lincRNAs and microRNAs

Thus far, it has been known that some lincRNAs play *cis* regulatory roles, and we were interested in whether some lincRNAs might have *trans* roles. Many lncRNAs play *trans* roles as competing endogenous RNAs (ceRNAs) to block the inhibitory regulation of microRNA (miRNAs) on mRNA targets [25–27].

To illustrate the interaction of lincRNAs and microRNAs, we also sequenced the microRNA expression profiles of *C. elegans* in the nine different stages and populations. A functional interaction network between lincRNAs and miRNAs was then built (Fig. 4g). We observed that of the 170 lincRNAs, 28 of them contained at least two miRNA seed regions in their sequences and showed a negative correlation with the corresponding microRNA at expression levels (Fig. 4g, Additional file 6: Table S4). Among these 28 lincRNAs, six, linc-22, linc-60, linc-73, linc-107, linc-109, and linc-126, showed phenotypes in this study (Figs. 2a and 4g). In fact, linc-109 was the lincRNA with the most microRNA interactions in this network.

**Fig. 5:**
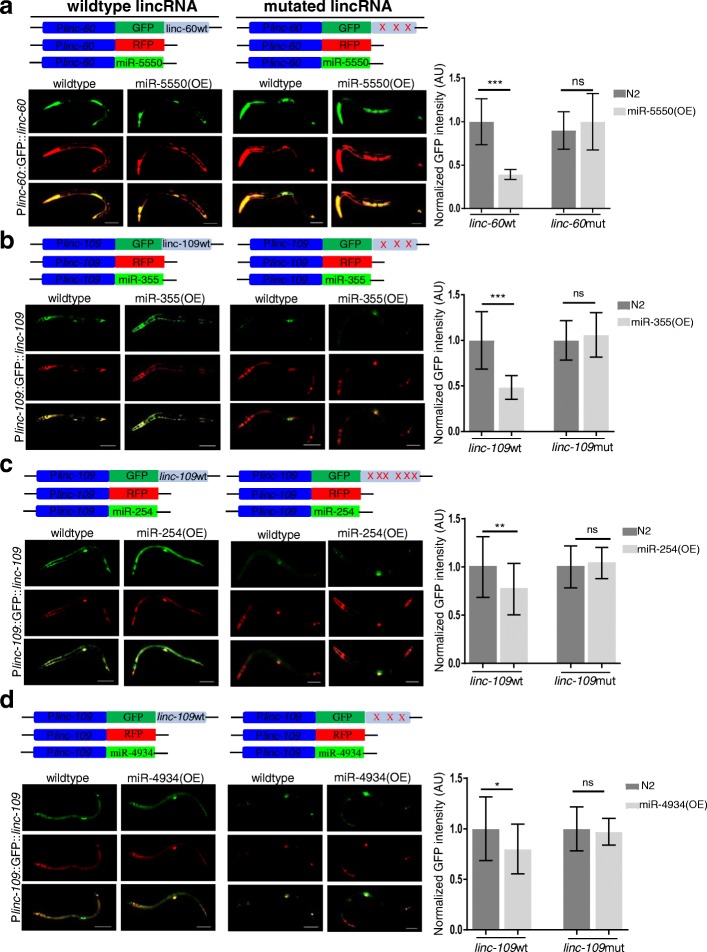
Regulation of lincRNAs by miRNAs. **a** Relative GFP expression level of *linc-60* in N2 worms with or without the overexpression of *mir-5550* (*n* = 20). **b** Relative GFP expression level of *linc-109* in N2 worms with or without the overexpression of *mir-355* (*n* = 20). **c** Relative GFP expression level of *linc-109* in N2 worms with or without the overexpression of *mir-254* (*n* = 20). **d** Relative GFP expression level of *linc-109* in N2 worms with or without the overexpression of *mir-4934* (*n* = 20). Constructs with mutations in the miRNA binding site of lincRNA were used as negative controls, and the positions of mutations are represented by red crosses. ns, no significance; *p < 0.05; **p < 0.01; ***p < 0.001; Student’s *t* test. Data are the means ± SD. Images shown are representative of the control and experimental groups. Scale bar, 20 μm

A dual-color system was used to determine the interaction of lincRNA-miRNA pairs in vivo, in which the 3′ UTR region of a GFP reporter was replaced with the complete sequences of the lincRNA of interest, and the corresponding lincRNA harboring the mutated microRNA binding sites was used as a negative control (Fig. 5). The relative GFP intensity of P*linc-60*::GFP::*linc-60* was stronger in N2 worms than mir-5550 overexpressing worms (Fig. 5a). *linc-109* was predicted to be regulated by 11 miRNAs (miR-5547-5p, miR-4805-5p, miR-1820-5p, miR-6208-5p, miR-8194-5p, miR-4934, miR-254-3p, miR-4814-3p, miR-355-5p, miR-5546-3p, and miR-239b-3p), and we examined 4 of the 11 miRNAs. P*linc-109*::GFP::*linc-109* showed weaker GFP expression in worms overexpressing mir-355, mir-254, or mir-4934 (Fig. 5b–d). However, another tested microRNA, miR-5546, had no effect on the expression of P*linc-109*::GFP::*linc-109* (Additional file 7: Figure S3a). Another predicted lincRNA and microRNA pair, linc-126 and mir-4938, also did not show an interaction in the dual-color in vivo assay (Additional file 7: Figure S3b). These results strongly indicated that certain lincRNAs could play *trans* roles as ceRNAs in *C. elegans*.

### Rescuing lincRNA phenotypes

**Fig. 6:**
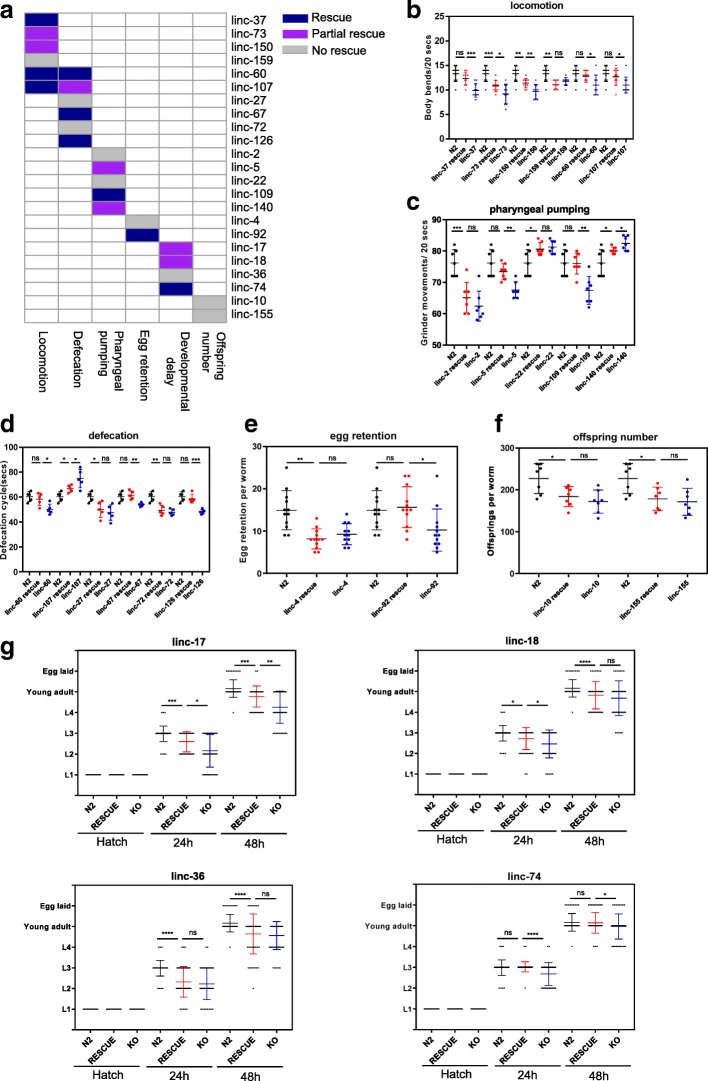
Rescuing lincRNA phenotypes. **a** Summary of the rescuing experiment results in all 23 lincRNA mutants. Rescue, the mutant phenotype was fully rescued. Partial rescue, the mutant phenotype was rescued, although data from the rescuing line were still significantly different from those of wild-type worms. **b** Rescuing data for the locomotion phenotypes in 6 lincRNA mutants. **c** Rescuing data for the pharyngeal pumping defects in 5 lincRNA mutants. **d** Rescuing data for the defecation defects in 6 lincRNA mutants. **e** Rescuing data for the decreased egg retention in 2 lincRNA mutant worms. **f** Rescuing data for the reduced number of progeny phenotype in 2 lincRNA mutants. **g** Rescuing data for the developmental delay in 4 lincRNA mutants. For b-g, the data for N2 worms were reused in the figure for comparison to the lincRNA mutants and rescue lines. For b-f, ns, no significance; *p < 0.05; **p < 0.01; ***p < 0.001; unpaired Student’s t-test. Data are the means ± SD. For **g**, ns, no significance; *p < 0.05; **, *p* < 0.01; *** *p* < 0.001; **** p < 0.0001; *p* values were calculated by the chi-square test

Rescue experiments can provide further insights into molecular mechanisms, and thus, we expressed the corresponding lincRNA with its own promoter in the 23 lincRNA mutants. Among these 23 lincRNA mutants, the phenotypes of 9 lincRNA mutants were fully rescued, those of 7 lincRNA mutants were partially rescued, and those of 9 lincRNA mutants were not rescued (Fig. 6a, Additional file 8: Table S5). Here, partial rescue meant that the rescuing line showed a statistically significant difference from the lincRNA mutants, although the defect was not fully recovered as data from the rescuing line were still significantly different from those of the wild-type worms. For locomotion defects, three lincRNA mutants, such as *linc-37*, could be fully rescued, two including *linc-73* could be partially rescued, and *linc-159* mutant was not rescued (Fig. 6b). For the other phenotypes in pharyngeal pumping, defecation, egg retention, offspring number, and developmental delay, we observed that two lincRNA mutants with defects in number of progenies could not be rescued with overexpression, and lincRNA mutants with one of the other four phenotypes could either be fully rescued, partially rescued, or not rescued (Fig. 6c–g). LincRNAs (e.g., linc-109) with phenotypes that could be fully rescued by overexpressing the corresponding lincRNA might mainly play *trans* roles, while those with phenotypes that could not be rescued by overexpressing the corresponding lincRNA (e.g., linc-27) might mainly play *cis* roles. LincRNAs (linc-73) with phenotype that could be partially rescued might possess both *trans* and *cis* roles. For phenotypes that likely link to germline expression (e.g., linc-10 and linc-155), failure to rescue might be due to silencing of the overexpressing extrachromosomal constructs. Of course, links between the rescuing result and the molecular mechanism might be more complex, and we were comparing it with other results.

### Transcriptional regulation of lincRNAs

**Fig. 7:**
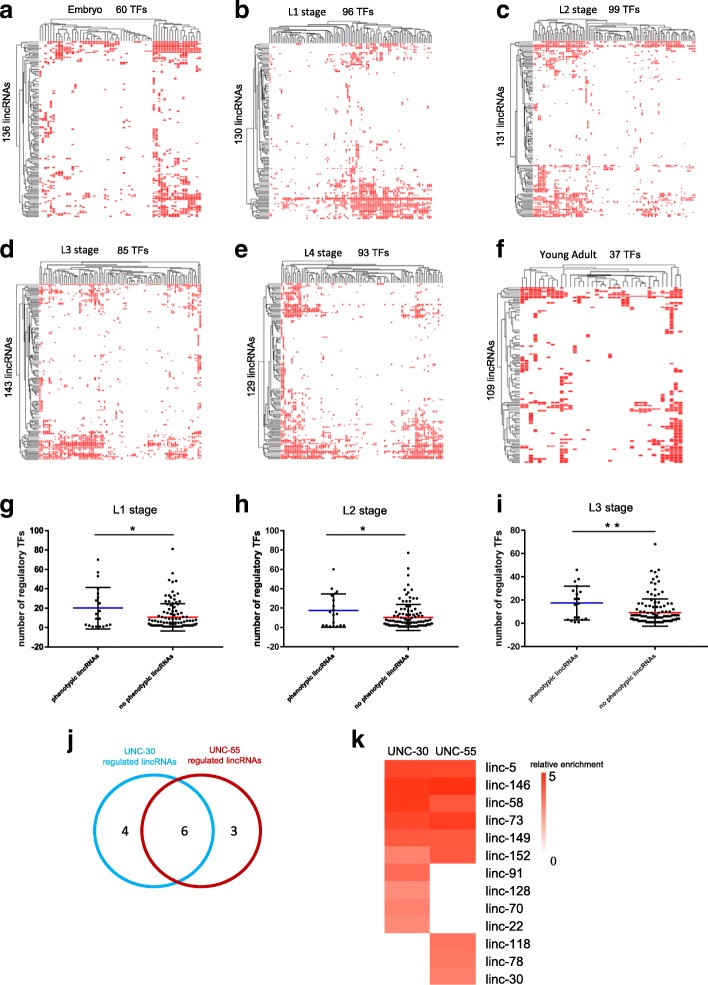
Regulation of lincRNAs by transcription factors. **a** Clustering map illustrating the binding of transcription factors to lincRNA genes in embryos. **b** Clustering map illustrating the binding of transcription factors to lincRNA genes in L1 stage. **c** Clustering map illustrating the binding of transcription factors to lincRNA genes in L2 stage. **d** Clustering map illustrating the binding of transcription factors to lincRNA genes in L3. **e** Clustering map illustrating the binding of transcription factors to lincRNA genes in L4 stage. **f** Clustering map illustrating the binding of transcription factors to lincRNA genes in young adult worms. **g–i** Number of transcription factors regulating the 23 lincRNAs with phenotypes in this study and the other 147 lincRNAs in L1 worms (**g**), L2 worms (**h**), and L3 worms (**i**). **j** UNC-30 and UNC-55 regulated lincRNA targets. **k** Heatmap of the relative ChIP-seq enrichment of UNC-30 and UNC-55 lincRNA targets. All data illustrated in a-f were downloaded from modENCODE. *p < 0.05; **, *p* < 0.01; *** *p* < 0.001; *p* values were calculated by two-sided Mann-Whitney *U* test

The transcriptional regulation of noncoding RNAs has not been clearly understood because most studies have focused on protein-coding genes [28, 29]. We analyzed the chromatin immunoprecipitation sequencing (ChIP-seq) data of ~ 300 transcription factors in *C. elegans* downloaded from modENCODE to examine their binding sites on the genomic sequences of lincRNAs in 6 different stages [30, 31]. According to our re-analyzed data, 60 of 79 transcription factors were found to regulate a total of 136 lincRNAs in the embryo (Fig. 7a); 96 of the 116 transcription factors showed binding to the genomic region of 130 lincRNAs in L1 stage (Fig. 7b); 99 of 107 transcription factors regulated 131 lincRNAs in L2 stage (Fig. 7c); 85 of 108 transcription factor genes at L3 stage regulated the transcriptional expression of 143 lincRNAs (Fig. 7d); 93 of 110 transcription factors might regulate of the expression of 129 lincRNAs at L4 stage (Fig. 7e); and 37 of 39 the transcriptional factors showed binding to 109 lincRNA genes in young adults (Fig. 7f). Interestingly, the 23 lincRNAs with a phenotype in this study were regulated by more transcription factors than the other 147 lincRNAs in L1, L2, and L3 worms (Fig. 7g–i), while there was no significant difference in the number of transcription factors regulating these two groups of lincRNAs in embryos, L4 worms, and young adults (Additional file 9: Figure S4).

Previous studies by our group and others have shown that two transcription factors, UNC-30 and UNC-55, work together to specify GABAergic DD and VD motor neurons (mns) in *C. elegans* [32–34]. Therefore, we analyzed the ChIP-seq data from endogenously expressed UNC-30 and UNC-55 for their lincRNA targets [32]. UNC-30 regulated 10 lincRNAs, and UNC-55 regulated 9 lincRNAs (Fig. 7j). UNC-30 and UNC-55 shared 6 lincRNA target genes (*linc-5*, *linc-58*, *linc-73*, *linc-146*, *linc-149*, and *linc-152*) (Fig. 7j, k, Additional file 10: Figure S5). The 6 shared lincRNA targets showed a higher relative enrichment in ChIP-seq compared with lincRNA targets that were regulated by either UNC-30 or UNC-55 alone (Fig. 7k). Among the shared lincRNA targets of UNC-30 and UNC-55, *linc-5* and *linc-73* had phenotypes of pharyngeal pumping and locomotion, respectively (Fig. 2a). Promoter reporters of *linc-5* and *linc-73* demonstrated that both lincRNAs were expressed in the head region and the D mns (Fig. 3a, b).

### Molecular mechanism of linc-73 in locomotion

**Fig. 8:**
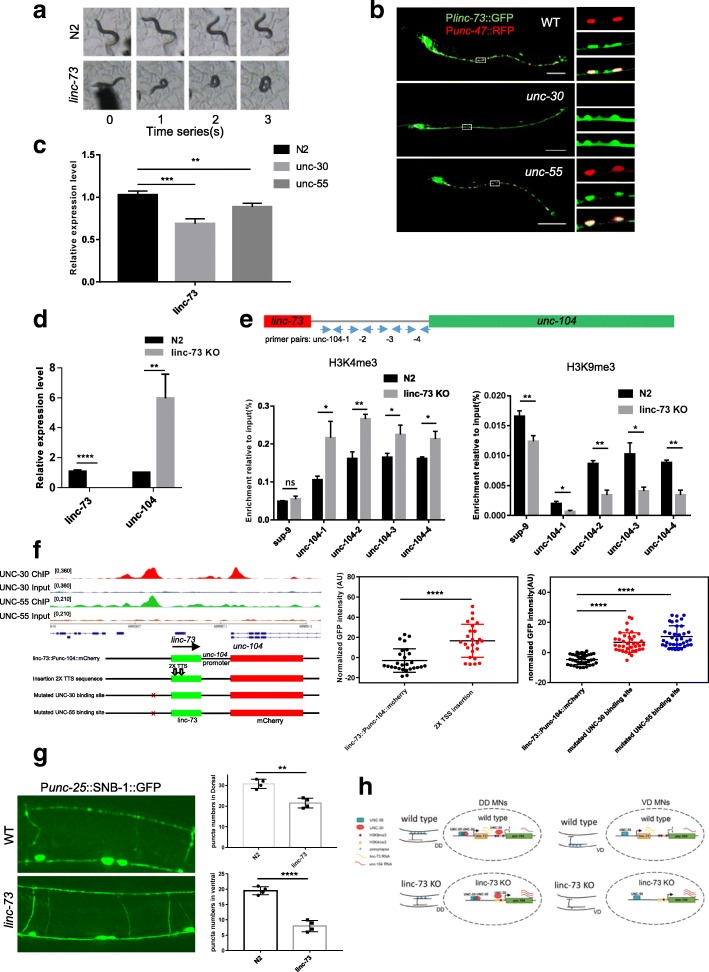
*cis* effect of *linc-73* on the neighboring gene *unc-104*. **a** Time-lapse observation of the uncoordinated backward movement of the *linc-73* CRISPR KO strain. **b** Expression pattern of *linc-73* in the wild-type, *unc-30*, and *unc-55* mutant backgrounds. Areas inside the dashed boxes are enlarged at the sides. P*unc-47*::RFP is a GABAergic marker. **c** qRT-PCR of *linc-73* RNA levels in L2 worms of N2, *unc-55(e1170)*, and *unc-30(e191).* **d** qRT-PCR of *unc-104* mRNA levels in N2 and *linc-73* KO worms (L2). **e** H3K4me3 (activation marker) and H3K9me3 (suppressive marker) at the promoter region of *unc-104* in N2 and *linc-73* mutants (L2 worms). The positions of the primer pairs used are indicated in the diagram. **f** Quantification of the relative expression levels of *unc-104::*mCherry in the cell body of D mns. Positions of the mutated UNC-30 (ΔUNC-30) & UNC-55 (ΔUNC-55) and insertion of the transcription terminal site (TTS) are shown along with the UNC-30 and UNC-55 ChIP-seq peaks. **g** Quantification of the dorsal and ventral presynaptic puncta (SNB-1::GFP) of DD mns in N2 and *linc-73* mutants (L2). Representative images are shown. **h** a working model for the regulation of UNC-30 & UNC-55 on linc-73, which then regulates the expression of *unc-104* to modulate *C. elegans* locomotion. *p < 0.05, **p < 0.01, ****p < 0.0001 by the Student’s *t* test. Scale bar, 50 μm

The *linc-73* CRISPR KO strain showed uncoordinated backward movement resulting in the formation of a ventral coil, which resembled the phenotype of the *unc-55* mutant (Fig. 8a). *linc-73* was expressed in GABAergic D mns and other cells (Fig. 8b), and its expression levels were decreased in either *unc-55(e1170)* or *unc-30(e191)* mutants (Fig. 8c). The decrease of *linc-73* expression in *unc-55(e1170)* or *unc-30(e191)* was mild, which could be explained by the expression of *linc-73* in cells without *unc-55* or *unc-30* expression. These results indicated that both UNC-30 and UNC-55 activated *linc-73* expression. The immediate downstream gene of *linc-73* was *unc-104*, a *C. elegans* kinesin gene [35–37], and the expression levels of *unc-104* were significantly increased in *linc-73* KO worms (Fig. 8d). We noticed that this change in expression levels was inconsistent with changes in H3K4me3 (activation marker) and H3K9me3 (suppressive marker) at the promoter region of *unc-104* when comparing the *linc-73* mutant to wild-type worms (Fig. 8e). When transcription terminal sites were inserted into the *linc-73* genomic region, the expression levels of *unc-104* were increased (Fig. 8f, Additional file 11: Figure S6). When the binding site of UNC-30 or UNC-55 in the promoter of *linc-73* was mutated, the expression levels of *unc-104* were also increased (Fig. 8f, Additional file 11: Figure S6). These results supported a model in which both UNC-30 and UNC-55 could activate the expression of linc-73 RNA, which played a *cis* role to modulate the histone epigenetic status of the *unc-104* promoter and therefore inhibit the expression of *unc-104*.

It is well known that *unc-104* plays essential roles in the transportation of presynaptic proteins [35–37]. There was a slight decrease in the dorsal presynaptic puncta for DD mns in *linc-73* mutants compared to a more dramatic decrease in the number of ventral presynaptic VD mn puncta (Fig. 8g). The detailed mechanism about how increased levels of UNC-104 in D mns resulted in asymmetric presynaptic punctum distribution remained for further investigation. These changes in DD and VD mns in *linc-73* mutants would result in relatively weaker inhibition of ventral vs dorsal body wall muscles in *linc-73* mutants and thus to a ventral coil phenotype. Taken together, these data suggested a model in which two transcription factors, UNC-30 and UNC-55, co-regulated the expression of *linc-73*, which then regulated the expression of *unc-104 in cis* by affecting histone modifications to modulate the formation of presynapses in the D mns and further to play roles in *C. elegans* locomotion (Fig. 8h).

## Discussion

LincRNAs are now recognized as critical players in eukaryotic cells [1–4]. Studies at the cellular level have uncovered a myriad of functions and functional mechanisms for many mammalian lincRNAs [7, 9, 38]. These lincRNAs can play roles either in the nucleus or in the cytoplasm with an array of *trans* and *cis* mechanisms [39, 40].

CRISPR enables fast and efficient genetic engineering, thus providing an opportunity to generate KO strains for nearly all the lincRNAs of an animal, *C. elegans*. Systematic analyses of these strains for just six traits identified 23 phenotypic lincRNAs; it would be reasonable to speculate that many lincRNAs or even most of them may be phenotypic lincRNAs given the analysis of more (or more complex) traits, such as chemosensory, longevity, and male mating. Researchers have just started to explore the roles of lincRNAs and other lncRNAs systematically with CRISPR screening in mammalian cell cultures [41–44]. LincRNAs do not have overlapping sequences with other genes, which makes them relatively more adaptive to perturbation, and the results from the manipulations are relatively easier to explain. Our understandings of lincRNAs could also be true for other lncRNAs, as lincRNAs have multiple features that are shared by many other lncRNAs. The study of lincRNAs and lncRNAs in *C. elegans* is relatively lagging behind that in mammalian cells. The *C. elegans* KO strains of lincRNAs from this study would be valuable resources for future studies, as this animal is a supreme model organism with powerful genetic and cell biology tools.

Critical roles of lincRNAs at the cellular level sometimes do not justify their physiological significance at the whole organismal level. For example, studies at the cellular level have demonstrated that MALAT1 plays major roles in nuclear speckles for mRNA processing, splicing, and export [45, 46]. However, there is no obvious phenotype in MALAT1 KO mice [47, 48]. Additionally, some recent arguments have been raised about the physiological roles of hotair, as some researchers believe that hotair KO mice do not show an apparent phenotype [49, 50]. Therefore, it is of great value to study lincRNAs both at the cellular level and with animals. Our lincRNA KO strains would facilitate studies at the whole organismal level. A pilot study using traditional method has generated KO strains for 18 murine lincRNAs, and essentially, all these mutants have phenotypes of embryonic lethal or severe defects in development leading to early death [51]. It is somewhat surprising that none of the 155 *C. elegans* lincRNA mutants have a lethal phenotype. It could be that the mammalian development is much more complicated, and the previous study also selected for lincRNAs with expression patterns of greater association with neural development [51].

To analyze the connections of *C. elegans* lincRNAs to other transcripts and epigenetic markers, we performed ChIP-seq of H3K4me3 and H3K9me3 for L4 worms and RNA-seq for both long RNAs (e.g., lncRNAs, mRNAs, and circular RNAs) and small RNAs (e.g., microRNAs) in nine worm developmental stages and populations (GSE115324). These are also valuable resources for future studies. Network construction and expression profile association can provide mechanism insights in the roles of lincRNAs. For example, co-expression analysis revealed that linc-109 was associated with muscle development and pharyngeal pumping, as well as microtubule-based movement (Fig. 4f), and the phenotype of *linc-109* mutant was a pharyngeal pumping defect. The lincRNA-microRNA co-expression and bioinformatic analyses revealed that linc-109 might be regulated by multiple microRNAs (Fig. 4g), and indeed, some of these regulatory effects were experimentally confirmed (Fig. 5). These points and the complete rescue of the *linc-109* phenotype by overexpressing this lincRNA (Fig. 6a, c) strongly suggested a *trans* regulatory role of linc-109, making it highly plausible that it serves as a ceRNA against microRNAs. lincRNAs can play *trans* roles other than ceRNA [39, 52, 53], and other potential trans roles of *C. elegans* lincRNAs require further investigations.

For the 8 lincRNAs that were expressed exclusively at one particular stage, only the linc-155 mutant had a phenotype, and the phenotype of a decreased number of progenies seemed to match its exclusive expression in early embryo (Figs. 1a, b and 2a, f). For the 12 lincRNAs that were ubiquitously expressed, only the *linc-4* mutant demonstrated a phenotype, egg retention (Figs. 1a, b and 2a, e), and it was difficult to speculate on any direct link between the ubiquitous expression of *linc-4* with the mutant phenotype. For the remaining 150 lincRNAs that were not expressed either ubiquitously or exclusively, the mutants of 21 lincRNAs showed phenotypes in the six traits examined (Figs. 1a, b and 2). For locomotion, defecation, pharyngeal pumping, egg retention, and offspring number, young adults were examined. Therefore, it was difficult to identify links between the corresponding expression pattern and the phenotype. For the four lincRNAs (linc-17, linc-18, linc-36, and linc-74) with a developmental delay, their mutants already did display retardation in early development within 24 h of hatching (Figs. 1a, b and 2a, g). All four of them showed relatively high expression levels in the embryo (Fig. 1a, b, Additional file 1: Table S1).

The expression of lincRNAs is under the control of transcription factors, and we noticed that a small portion (8 of ~ 300) of transcription factors (LIN-39, EOR-1, BLMP-1, NHR-77, HLH-1, DAF-16, W03F9.2, and NHR-237) regulated the expression of ≥ 50 lincRNAs (Fig. 7a–f). It would be interesting to further investigate the biological relevance underlying this regulatory phenomenon. A lincRNA can be transcriptionally regulated by multiple transcription factors together (Fig. 7). For example, lincRNA-73 is regulated by 48 transcription factors, including UNC-30 and UNC-55, two transcription factors that converge to control the differentiation and plasticity of GABAergic D mns [32–34]. Six lincRNAs are co-regulated by UNC-30 and UNC-55 (Fig. 7j) [23]. It was surprising that CRISPR knockout of only one of the six lincRNAs, *linc-73*, gave rise to uncoordination (Figs. 2a, b and 8). It is known how *linc-73* plays a cell-autonomous role in D mns to regulate the expression of *unc-104* (Fig. 8), but the roles of the other 5 lincRNAs that are also commonly regulated by UNC-30 and UNC-55, and why KO strains of these lincRNAs do not show a locomotion defect, remain to be elucidated. The 23 lincRNAs with mutant phenotypes in this study tended to be regulated by more transcription factors in L1, L2, and L3 worms (Fig. 7g–i). It is possible that these lincRNAs are related to greater physiological regulation, and thus, their perturbation may be more likely to cause defects. As for the regulation by histone modifications, our results show that both H3K4me3 and H3K9me3 regulate *linc-73* at L2 stage, although only H3K4me3 but not H3K9me3 binds to *linc-73* at L4 stage (Figs. 1c and 8e). H3K9me3 does not have that many genomic binding peaks as compared to H3K4me3 in our study and also in data from others (NCBI BioProject: PRJEB20485).

We have presented data to support that linc-73 plays a *cis* role to regulate the expression of *unc-104* (Figs. 4a and 8), although it is possible that linc-73 also has a *trans* role because the overexpression of linc-73 via an extrachromosomal construct could partially rescue the *linc-73* phenotype (Fig. 6a, b). However, linc-109 has been shown to function with *trans* roles (Figs. 4g, 5b–d, and 6a, c), although the expression of neighboring genes is altered in *linc-109* KO, which may be an indication of a *cis* role (Fig. 4a). The effects of *linc-109* KO on the expression of its neighboring genes may not contribute to the mutant phenotype, as the extrachromosomal construct could fully rescue the *linc-109* phenotype (Fig. 6a, c). The application of CRISPR actually deletes the DNA sequences of lincRNAs, which may harbor DNA elements that regulate the expression of neighboring genes. Thus, for each individual lincRNA, an array of experiments must be performed to elucidate the potential *cis* and/or *trans* role.

## Conclusions

By using CRISPR, we have generated knockout strains of 155 *C. elegans* lincRNAs as valuable resources for studies in ncRNAs. Systematic analyses of these strains for just six traits identified phenotypes in 23 lincRNA mutants. We have characterized some aspects of the expression patterns, molecular mechanisms, and other regulatory relevance of these lincRNAs.

## Methods

### Animal cultures and strains

Unless otherwise stated, all *C. elegans* strains used in this study were maintained on standard nematode growth medium (NGM) at 20 °C or 25 °C [54]. N2 Bristol was obtained from the Caenorhabditis Genetic Center (CGC). Eight strains including XIL0375, XIL0389, XIL1172, XIL0354, XIL1177, XIL0386, XIL0411, and XIL1237 were gifts from Dr. Xiao Liu. All worm strains generated or used in this study are listed in Additional file 3: Table S2.

### Worm synchronization

Gravid adult worms were washed three times with M9 and collected into 1.5 ml tubes, after which the tubes were centrifuged at 600 g. Animals were then treated with hypochlorite. Synchronized embryos were cultured at 20 °C on NGM plates with seeded OP50.

### Plasmid construction

pDD162, expressing Cas9 II protein, was a kind gift from Dr. Guangshuo Ou. For long lincRNAs (> 2 kb), 3–6 sgRNAs were designed to target the 5′ ends of the lincRNA. In the case of short lincRNAs (< 2 kb), 2–3 sgRNAs targeting the 5′ and 3′ ends of each lincRNA were used. In order to enhance the efficiency of the sgRNA, we specifically selected sgRNAs containing two NGG PAM motif in the 3′ ends of sgRNA sequence. All the sgRNA sequence used in this work were assessed at http://crispor.tefor.net/. The 20 nt sgRNA sequence was inserted behind the U6 promoter of pPD162 plasmid between the EcoRI and HindIII restriction endonuclease sites. Homology recombination plasmids were generated by cloning the 1.5 kb DNA sequence upstream of the site of interest, 2 kb lincRNA promoter sequence, GFP sequence, and 1.5 kb DNA sequence downstream of the site of interest between Sph I and Apa I of the pPD117.01. For lincRNA transcriptional reporters, approximately 2.5 kb promoter sequence was cloned from genomic DNA. The corresponding product was fused with sl2 sequence and was inserted between the Sph I and Age I of pPD117.0 expressing GFP or between Pst I and Age I of pPD95.67 expressing RFP (Andrew Fire collection, Addgene). For rescue plasmids, 2 kb promoter sequence was cloned from the genomic DNA, and lincRNA full-length sequence was cloned from cDNA. All those products were inserted into the pPD117.01 between the Sph I and Apa I double-digested sites. In the dual-color system for the in vivo analysis of miRNA-lincRNA interaction, we constructed GFP reporters for the selected lincRNA by replacing the 3′ UTR region of pPD117.01 with the complete wild-type sequence of the lincRNA of interest. As a control, the mutated versions of each lincRNA, in which the respective miRNA binding sites within the lincRNAs were mutated, was also cloned into pPD117.01. miRNA overexpression plasmids were constructed cloning the pri-miRNA sequence of the miRNA into pPD95.67 driven by promoter of the corresponding lincRNA. For linc-73::Punc-104::mCherry plasmid, linc-73 (TTS insertion)::Punc-104::mCherry plasmid and linc-73 (mutated UNC-30 binding site)::Punc-104::mCherry plasmid construction, linc-73 promoter and gene body sequence, unc-104 promoter sequence, mCherry sequence were cloned separately and inserted into the pPD117.01. The UNC-30 or UNC-55 binding site in linc-73 promoter was mutated from GATTA to CTCAG (for UNC-30) or from ATCGATCCAT to CGATCGAACG (for UNC-55). 2X transcriptional terminal site (2X TTS, AAATAAAATTTTCAGAAATAAAATTTTACA) was inserted into the 5′ portion of linc-73. A list of primers used is provided in Additional file 12: Table S6.

### Injection of CRISPR/Cas9 knockout and knock-in and other plasmids

CRISPR/Cas9 system was carried out as previously described with modifications [23]. For the knockout system, we mixed 3–6 Pu6::lincRNA sgRNA plasmids (30 ng/μl of each) and pPD162 expressing Cas9 II protein (30 ng/μl), as well as co-injection marker P*myo-2*::mCherry (PCFJ90) (10 ng/μl) together. The mixture was injected into about 30 N2 adults (adulthood day 1). For the CRISPR knock-in, the upstream locus of *linc-1* in chromosome I was selected as the knock-in site due to the presence of fewer genes located in the *linc-1* neighborhood. PU6::sgRNA plasmids (30 ng/μl), PPD162 plasmid (30 ng/μl), co-marker plasmid (10 ng/μl), and homologous recombination plasmid (40 ng/μl) were injected into the 30 gravid worms, and transgenes were selected as described above. All the knockout or knock-in mutant worms were transferred to new plates and outcross for at least three generations to eliminate off targets. In the dual-color system, wild-type or mutated lincRNA reporters (20 ng/μl) were mixed with miRNA overexpression plasmids (20 ng/μl), control plasmids (20 ng/μl), and a 1-kb DNA ladder (Invitrogen) standard. For rescue experiment, overexpression plasmid of lincRNAs (20 ng/μl) was mixed with co-maker plasmid (PCFJ90 20 ng/μl) as well as DNA ladder (Invitrogen). linc-73::Punc-104::mCherry plasmid (20 ng/μl), linc-73 (TTS insertion)::Punc-104::mCherry plasmid (20 ng/μl), linc-73 (mutated UNC-30 binding site)::Punc-104::mCherry plasmid (20 ng/μl)and linc-73 (mutated UNC-55 binding site)::Punc-104::mCherry plasmid (20 ng/μl) was mixed with myo-2::GFP separately, and injected into gravid young adults. Standard microinjection techniques were used.

### Screening for CRISPR deletion and knock-in strains

Approximately, 200 F1 worms were singled after the injection and cultured at 25 °C. Genomic DNA of the F3 generation was extracted and examined by PCR. Worms were harvested and transferred to 100 μl lysis buffer (20 μg/ml Proteinase K, 100 mM KCl, 10 mM PH8.3 Tris-HCl, 1.5 mM MgCl2), and then placed at − 80 °C for 10 mins, thawed at 65 °C for at least 2 h. Worms were then placed at 95 °C for 15 mins to inactivate proteinase K, and 2 μl each worm lysate was used as DNA template for PCR amplification with primers spanning sgRNA-targeted regions. For the verification of the knock-in strains, we amplified genomic regions spanning the point of insertion. Worms with the corrected PCR products were singled to NGM plates and further confirmed by DNA sequencing of the genomic PCR products. CRISPR worms were outcrossed at least three times before being used in experiments. The primers used for PCR screening are listed in Additional file 12: Table S6.

### Locomotion

To examine locomotion of worms, young adult worms were removed from the bacterial lawn of an agar culture plate to bacteria-free plates at room temperature, and allowed to crawl away from any food remains for about 10–20 s. Complete body bends per 20 s were then counted under a dissecting microscope after animals were gently touched at the tail end (*n*, number of worms = 5; *N*, number of replicates = 3) [55].

### Defecation assay

Defecation cycles were performed according to previous report [56]. Data was presented by recording the time between defecation cycles of young adult worms (*n*, number of worms = 5; *N*, number of replicates = 3).

### Pharyngeal pumping

Pharyngeal pumping behavior was assayed as previously described [55, 57]. Pharyngeal pumping was examined by counting grinder movements for 20 s at 20 °C (*n*, number of worms = 7; *N*, number of replicates = 3).

### Egg retention

Egg retention assay was carried out as described earlier with some modifications [58]. One day (post the last molt) old adult worms were singled out and lysed in hypochlorite solution for 6 mins in 96-well plate, and the number of eggs was counted (*n*, number of worms = 12; *N*, number of replicates = 3).

### Examination of development stages

To examine the development stages of worms, synchronized eggs were allowed to hatch at 20 °C and allowed to grow at NGM plates with adequate food and their developmental stages were examined after 24 h and 48 h (*n*, number of worms = 30; *N*, number of replicates = 3) [59].

### Number of progenies

L4 worms were singled on NGM plates and allowed to lay eggs at 20 °C [60]. Individual worms were transferred daily from the start of egg laying until egg laying stopped. The number of live offspring (L1) were counted (*n*, number of worms = 7; *N*, number of replicates = 3). All experiments were performed under a dissecting microscope.

### Quantitative RT-PCR (qRT-PCR) and quantitative PCR (qPCR)

RNAs were extracted from worms in TRIzol L/S solution (Invitrogen) after three cycles of freezing at − 80 °C and thawing at room temperature. Five hundred nanogram total RNAs were reverse transcribed into cDNA by cDNA synthesis kit (Goscript™ Reverse Transcription System, Promega). qRT-PCR (with cDNA template) and qPCR (with genomic DNA template) were performed using a GoTaq qPCR Master Mix kit (Promega) on a PikoReal 96 real-time PCR system (Thermo Scientific) according to standard procedures. 18S RNA was used for normalization. All PCR products were sequenced for confirmation. All primers used are listed in Additional file 12: Table S6.

### Microscopy and calculating the relative fluorescence intensity

For all the lincRNAs reporter worms, Axio Scope A1 compound microscope (Zeiss, Oberkochen, Germany) was used for the examination of fluorescence. L4 stage worms were anesthetized in 10 mM sodium azide, and images were taken using the × 20 objective. All the images were analyzed by the ImageJ (an open-source image processing software). Confocal imaging was carried out as previously reported with some modification [32]. Imaging of anesthetized worms were carried out on Andor Revolution XD laser confocal microscope system (Andor Technology PLC) based on a spinning-disk confocal scanning head CSU-X1 (Yokogawa Electric Corporation) under control of Andor IQ 10.1 software or two-photon confocal laser scanning microscopy FV1200MPE (Olympus) with GaAsP-NDD detector. Z-stack images were obtained on Olympus IX-71 inverted microscope (Olympus Corporation) with × 60 1.45 NA oil-immersion objective. An Andor iXonEM+ DV897K EM CCD camera was used for capturing the 14-bit digital images with Andor LC-401A Laser Combiner with diode-pumped solid state (DPSS) lasers, emissions at 458 nm, 488 nm, 515 nm, and 561 nm.

### Counting the presynaptic puncta in ventral and dorsal

Dorsal nerve cord and ventral nerve cord images were obtained and counted between VD9 and VD11. ImageJ plot profile tool was used to plot nerve cords, and the number of SNB-1::GFP (Punc-25::snb-1::gfp) puncta was calculated by counting the number of crests of the plot file (*n* = 4).

### RNA sequencing

For next-generation RNA sequencing, total RNAs were isolated from nine different stages of worms (embryos, L1, L2, dauer, L3, L4, young adult, male, and mix stage with starvation). Sequencing libraries were carried out as previously described with modifications [53]. Whole transcriptome libraries were constructed by the TruSeq Ribo Profile Library Prep Kit (Illumina, USA), according to the manufacturer’s instructions. In brief, 10 μg total RNA was depleted rRNA with an Illumina Ribo-Zero Gold kit and purified for end repair and 5′-adaptor ligation. Then, reverse transcription was performed with random primers containing 3′ adaptor sequences and randomized hexamers. The cDNAs were purified and amplified, and PCR products of 200–500 bp were purified, quantified, and stored at − 80 °C until sequencing. For RNA sequencing of long RNAs, the libraries were prepared according to the manufacturer’s instructions and subjected to 150 nt paired-end sequencing with an Illumina Hiseq 2500 system (Novogene, China). We sequenced each library to a depth of 10–50 million read pairs, and the reads were mapped to the *C. elegans* genome (ce11). For small RNA (sRNA) sequencing, nine sRNA libraries were generated with TruSeq small RNA (Illumina, USA) according to the manufacturer’s instructions. Then, the prepared libraries were sequenced with an Illumina Nextseq 500 system (Novogene, China). After filtering out the reads shorter than 15 nt, the remaining reads were mapped to the *C. elegans* genome (ce11) and the miRNA database in miRBase with bowtie (-v 1).

### Conservation, length, and exon number analysis of lincRNAs

For the genome-wide feature analysis of lincRNAs, the control was 200 mRNAs randomly picked from *C. elegans* transcriptome. The information of length and exon number for lincRNAs and mRNAs was extracted from the annotation of *C. elegans* [61]. For the analysis of sequence conservation, we interrogated 26 nematode conservation phastCons scores from UCSC [61] for each base of individual *C. elegans* lincRNA or mRNA and averaged the scores of each transcript. The distribution of lincRNA and mRNA was compared by two-sided Mann-Whitney *U* test.

### Construction of lincRNA-miRNA co-expression network

Functional networks of miRNA and lincRNA pairs were illustrated with cytoscape v3.5.1 [62]. For the one-to-one connection, the expression of lincRNA with at least two 7-mers matches of particular miRNA was negatively correlated to the expression of miRNA (Pearson *R* < − 0.1) across nine stages.

### Chromatin immunoprecipitation (ChIP)

ChIP assays were performed as described in our previous report with modifications [23]. N2 and linc-73 mutant worms were bleached with hypochlorite solution, and the eggs were incubated at 20 °C on NGM plates seeded with OP50 to be synchronized to L2 (for ChIP-qPCR experiments) or L4 stage (for ChIP-seq experiments). Synchronized worms were then washed with three changes of M9 buffer and fixed with 2% formaldehyde for 35 min followed by stopping with 100 mM Tris pH 7.5 for 2 min. Worm pellets were washed with FA buffer supplemented with 10 μl 1 M DTT, 50 μl 0.1 M PMSF, 100 μl 10% SDS, 500 μl 20% N-Lavroyl sarcosine sodium, and 2 tablets protease inhibitors in 10 ml FA buffer. Worms were sonicated on ice for 15 min with the setting of high power, 4 °C, and 15 cycles, 30 s on, 30 s off. The tubes were then spun at 14,000 g for 10 min at 4 °C. The supernatant was carefully removed into new tubes, and an aliquot (5% of each sample) was taken as input. Prewashed salmon sperm Protein G beads were added to the supernatant for 1 h for pre-cleaning. Beads were discarded, and 2 μg anti-H3K4me3 or anti-H3K9me3 (Abcam) were added to each tube overnight at 4 °C. The beads were washed twice with 150 mM NaCl FA buffer for 5 min each, washed once with 1 M NaCl FA buffer for 5 min, twice with 500 mM NaCl FA buffer for 10 min, once with TEL buffer (0.25 M LiCl, 1% NP-40, 1% sodium deoxycholate, 1 mM EDTA, 10 mM Tris-HCl, pH 8.0) for 10 min, and finally with three changes of 1X TE buffer (1 M Tris-HCl, 0.5 M EDTA). DNA-protein complexes were eluted in 200 μl of ChIP elution buffer (1% SDS in TE with 250 mM NaCl) and incubated at 65 °C for 20 min with regular shaking every 5–10 min. Both samples and inputs were treated with RNase A (2 μg/μl) and proteinase K (2 μg/μl) for 2 h at 55 °C for 1 h and then reverse cross-linked at 65 °C overnight. DNA was purified by phenol/chloroform/isoamyl extraction and then used for ChIP-qPCR or ChIP-seq. For ChIP-seq, DNA from ChIP (along with the input) was iron fragmented at 95 °C followed by end repair and 5′ adaptor ligation, then purified and amplified. PCR products corresponding to 200–500 bps were purified for sequencing. Illumina Nextseq 500 system for 150 nt pair-end sequencing was then performed (Novogene).

### Analysis of ChIP-seq data of transcription factors and H3K4me3 and H3K9me3

A total of 774 ChIP-seq raw fastq data and 561 computed gff3-file data were downloaded from modENCODE (ftp://data.modencode.org/C.elegans/Transcriptional-Factor/ChIP-seq/) [30, 31], and the regulation patterns of all transcriptional factor genes by lincRNAs in *C. elegans* were analyzed. The quality of all these 774 raw fastq data was verified using bowtie2 to map the reads to the *C. elegans* genome (ce11). We then re-analyzed the calculated peaks in order to investigate the regulation of transcriptional factors by the various lincRNAs. Considering the shorter length of the lincRNAs transcripts as compared to the mRNAs, we used the scale within 1 kb upstream or 200 bp downstream of the transcription start site of the lincRNAs. ChIP-seq data of UNC-30::GFP and UNC-55:GFP from our previous study using endogenous GFP knock-in *unc-30* and *unc-55* mutant worms were also analyzed (GEO: GSE102213) [23]. Reads were first filtered from genomic repeats, and the unique reads were then mapped to the *C. elegans* genome (ce11) with bowtie2. Peaks of UNC-30 and UNC-55 were assigned by the cisGenome with default parameters (cutoff > 3 and *p* value < 10^−5^). H3K4me3 and H3K9me3 ChIP-seq data of L4 were mapped to the *C. elegans* genome (ce11) with bowtie2 using the default parameters. Samtools were used to filter *sam files and remove duplicated reads. Macs2 was used for peak calling (q parameter was set as 0.001).

### Short Time-series Expression Miner analysis (STEM)

The co-expression patterns of lincRNAs and mRNAs were calculated by STEM [2 (a software program designed for clustering comparing, and visualizing gene expression data from short time series experiments) using RNA-seq data from nine different developmental stages. RNA-seq data from the embryonic stage was set as 0 point, and the other developmental stages were normalized to the embryonic stage data. *K*-means method was used to cluster the genes into specific profile according to their expression pattern. In all, nearly 20,000 genes with reads per kilo million (RPKM) greater than 1 were clustered into 10 profiles according changes in their expression patterns at different stages of development. The function of genes in specific clusters with similar expression patterns was analyzed by gene ontology analysis.

### GO analysis

The significant enriched genes were analyzed with Gorilla web-server [63]. *P* values were calculated with default parameters.

### Statistical analysis

For Student’s *t* tests, the values reported in the graphs represent averages of independent experiments, with error bars showing s.e.m. in all figures, except for Fig. 5 and Additional file 7: Figure S3, in which error bars show S.D. Statistical methods are also indicated in the figure legends. All statistical significances were determined using GraphPad Prism software (version 7). Two-sided Mann-Whitney *U* test was used in Figs. 1d, e and 7g–i and Additional file 7: Figure S3. Unpaired Student’s *t* test was used in Figs. 2b–f, 5, 6b–f, and 8c–g and Additional file 7: Figure S3. Chi-square test was used in Figs. 2g and 6g.

## Additional files

Additional file 1: **Table S1.** Expression profiles of lincRNAs. (XLSX 68 kb)

Additional file 2: **Figure S1.** Schematic diagram of the CRISPR/cas9 knockout system used in this study. (DOCX 117 kb)

Additional file 3: **Table S2.** List of worm strains. (XLSX 20 kb)

Additional file 4: **Table S3.** Data for the lincRNA mutant phenotypes. (XLSX 66 kb)

Additional file 5: **Figure S2.** Correlation of the expression levels of lincRNAs and their neighboring protein-coding genes. a Relative expression levels of 170 *C. elegans* lincRNAs and their neighboring mRNAs (100 kb upstream and downstream of the lincRNA locus). b Relative expression levels of 23 lincRNAs with distinct phenotypic characteristics and their neighboring mRNAs (100 kb upstream and downstream of the lincRNA locus) (DOCX 111 kb)

Additional file 6: **Table S4.** LincRNA-microRNA interactions. (XLSX 10 kb)

Additional file 7: **Figure S3.** miRNA regulation of lincRNAs. **a** Relative GFP expression levels of *linc-126* in N2 worms with or without overexpression of miR-4938 (*n* = 20). **b** Relative GFP expression levels of *linc-109* in N2 worms with or without overexpression of miR-5546 (n = 20). Data are the means ± SD. ns, not significant by the Student’s t-test. Images shown are representative of the control and experimental groups. Scale bar, 20 μm (DOCX 84 kb)

Additional file 8: **Table S5.** Data for the rescue experiments. (XLSX 12 kb)

Additional file 9: **Figure S4.** Numbers of transcription factors regulating the 23 lincRNAs with phenotypes in this study and the other 147 lincRNAs in embryos (**a**), L4 worms (b), and young adults (c). ns, no significant difference, by the two-sided Mann-Whitney U test (DOCX 46 kb)

Additional file 10: **Figure S5.** Shared UNC-30 and Unc-55 lincRNA targets with peak patterns in both UNC-30 and UNC-55 ChIP-seq. a *linc-5*, b *linc-58*, c *linc-146*, d *linc-149*, e *linc-152*.. (DOCX 75 kb)

Additional file 11: **Figure S6.** Expression of P*unc-104::*mCherry in the cell body of D mns. Representative images of data presented in Fig. 8f. Scale bar, 25 μm (DOCX 100 kb)

Additional file 12: **Table S6.** Primers used in this study. (XLSX 39 kb)

## Abbreviations

*C. elegans*: *Caenorhabditis elegans*
ChIP-seq: Chromatin immunoprecipitation sequencing
CRISPR: Clustered regularly interspaced short palindromic repeats
D mns: D motor neurons
GO: Gene ontology
KO: Knockout
lncRNA: Long intergenic noncoding RNA
PRC2: Polycomb repressive complex2
RPKM: Reads per Kilobase per Million mapped reads
