# Supplementary material for: Systematic evaluation of C. elegans lincRNAs with CRISPR knockout mutants
Source: Genome Biol. 2019 Jan 8;20:7. doi: 10.1186/s13059-018-1619-6 (PMC6325887; doi:10.1186/s13059-018-1619-6)
Supplement: Supplementary file 1 [file 13059_2018_1619_MOESM10_ESM.docx]

Figure S5

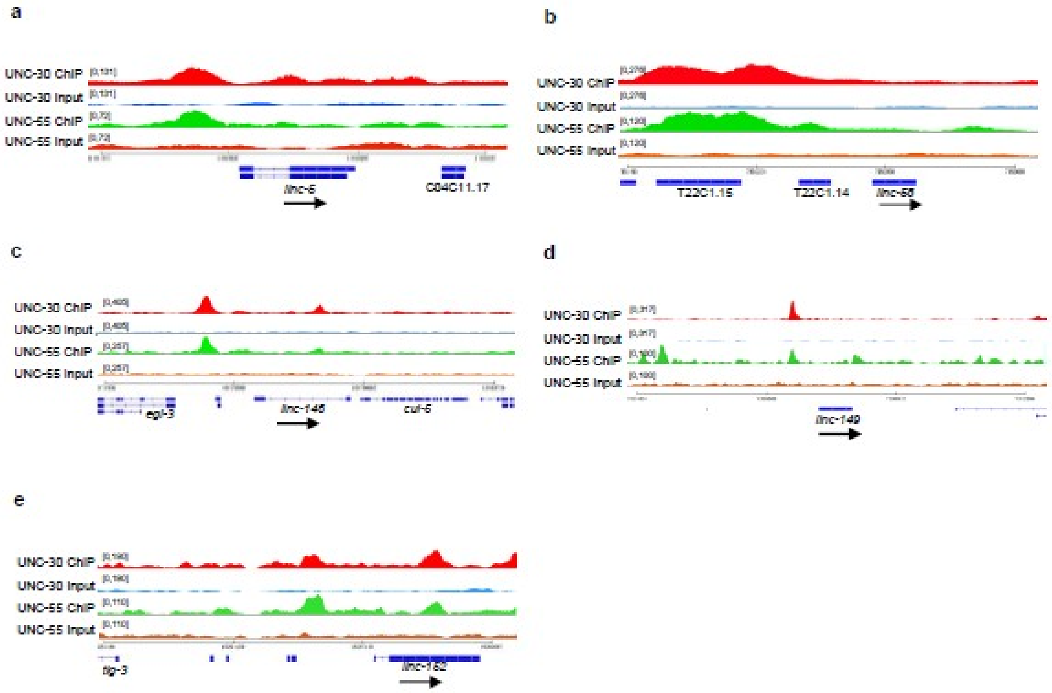
a
UNC-30 ChIP
UNC-30 Input
UNC-55 ChIP
UNC-55 Input
C04C11.17
b
UNC-30 ChIP
UNC-30 Input
UNC-55 ChIP
UNC-55 Input
T22C1.15
T22C1.14
c
UNC-30 ChIP
UNC-30 Input
UNC-55 ChIP
UNC-55 Input
egl-3
linc-146
cul-5
d
UNC-30 ChIP
UNC-30 Input
UNC-55 ChIP
UNC-55 Input
linc-149
e
UNC-30 ChIP
UNC-30 Input
UNC-55 ChIP
UNC-55 Input
tig-3
linc-152
