# Supplementary material for: Systematic evaluation of C. elegans lincRNAs with CRISPR knockout mutants
Source: Genome Biol. 2019 Jan 8;20:7. doi: 10.1186/s13059-018-1619-6 (PMC6325887; doi:10.1186/s13059-018-1619-6)
Supplement: Supplementary file 2 [file 13059_2018_1619_MOESM11_ESM.docx]

Figure S6

a

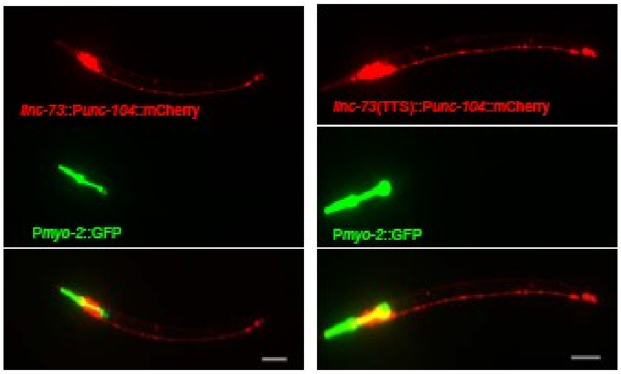

llnc-73::Punc-104::mCherry
Pmyo-2::GFP
llnc-73(TTS)::Punc-104::mCherry
Pmyo-2::GFP

b

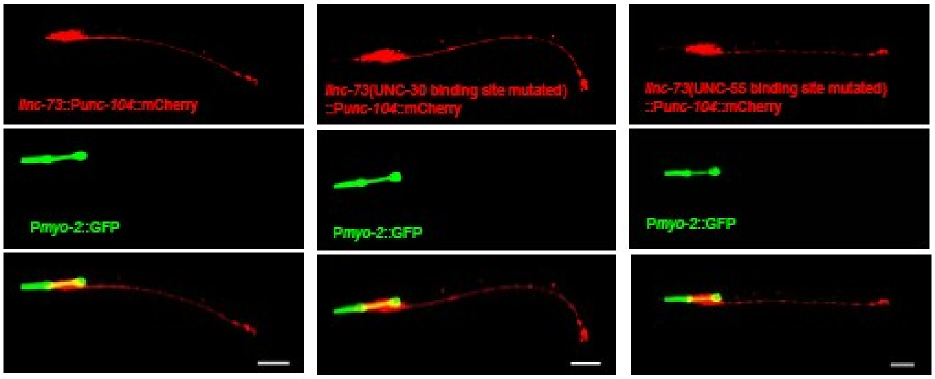

llnc-73::Punc-104::mCherry
Pmyo-2::GFP
llnc-73(UNC-30 binding site mutated)
::Punc-104::mCherry
Pmyo-2::GFP
llnc-73(UNC-55 binding site mutated)
::Punc-104::mCherry
Pmyo-2::GFP
