# Supplementary material for: Systematic evaluation of C. elegans lincRNAs with CRISPR knockout mutants
Source: Genome Biol. 2019 Jan 8;20:7. doi: 10.1186/s13059-018-1619-6 (PMC6325887; doi:10.1186/s13059-018-1619-6)
Supplement: Supplementary file 3 [file 13059_2018_1619_MOESM2_ESM.docx]

Figure S1

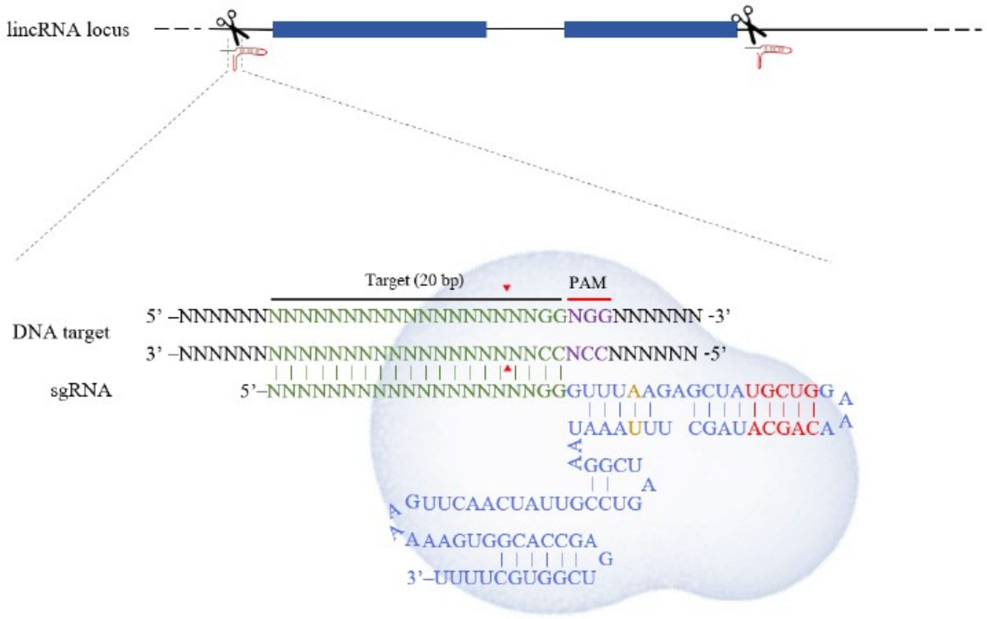

lincRNA locus
Target (20 bp)
PAM
DNA target
5' –NNNNNNNNNNNNNNNNNNNNNNNNNNGGNGGNNNNNN -3'
3' –NNNNNNNNNNNNNNNNNNNNNNNNNNCCNCCNNNNNN -5'
sgRNA
5'–NNNNNNNNNNNNNNNNNNNNGGGUUUAAGAGCUAUGCUGG
UAAAUUU CGAUACGACA
GGCU
GUUCAACUAUUGCCUG
AAAGUGGCACCGA
3'–UUUUCGUGGCU
