# Supplementary material for: Systematic evaluation of C. elegans lincRNAs with CRISPR knockout mutants
Source: Genome Biol. 2019 Jan 8;20:7. doi: 10.1186/s13059-018-1619-6 (PMC6325887; doi:10.1186/s13059-018-1619-6)
Supplement: Supplementary file 4 [file 13059_2018_1619_MOESM5_ESM.docx]

Figure S2

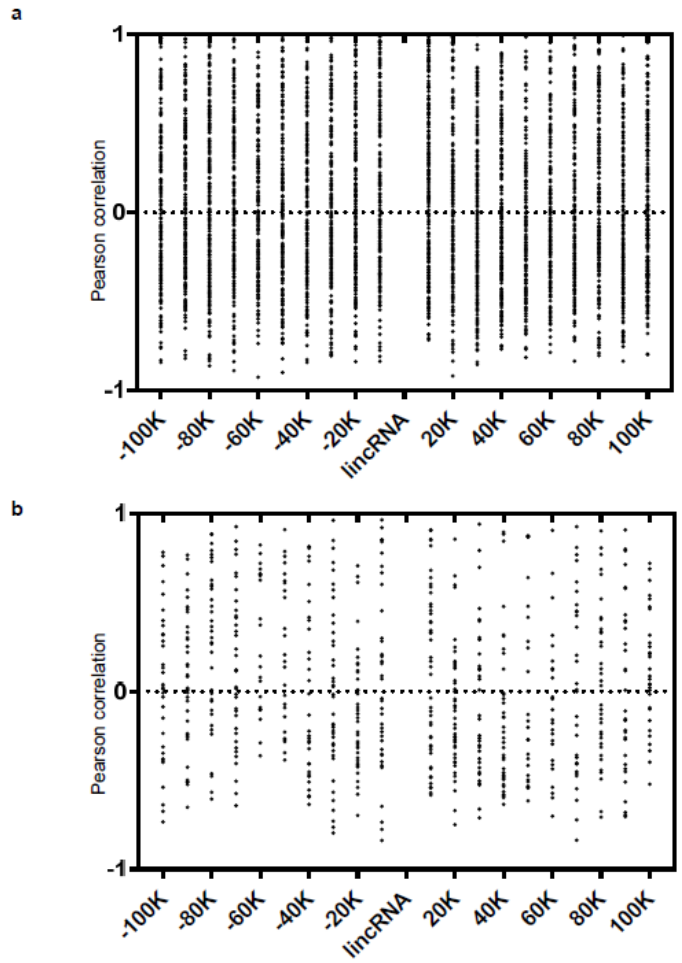
a
1
0
-1
Pearson correlation
-100K
-80K
-60K
-40K
-20K
lincRNA
20K
40K
60K
80K
100K
b
1
0
-1
Pearson correlation
-100K
-80K
-60K
-40K
-20K
lincRNA
20K
40K
60K
80K
100K
