# Supplementary material for: Systematic evaluation of C. elegans lincRNAs with CRISPR knockout mutants
Source: Genome Biol. 2019 Jan 8;20:7. doi: 10.1186/s13059-018-1619-6 (PMC6325887; doi:10.1186/s13059-018-1619-6)
Supplement: Supplementary file 5 [file 13059_2018_1619_MOESM7_ESM.docx]

Figure S3

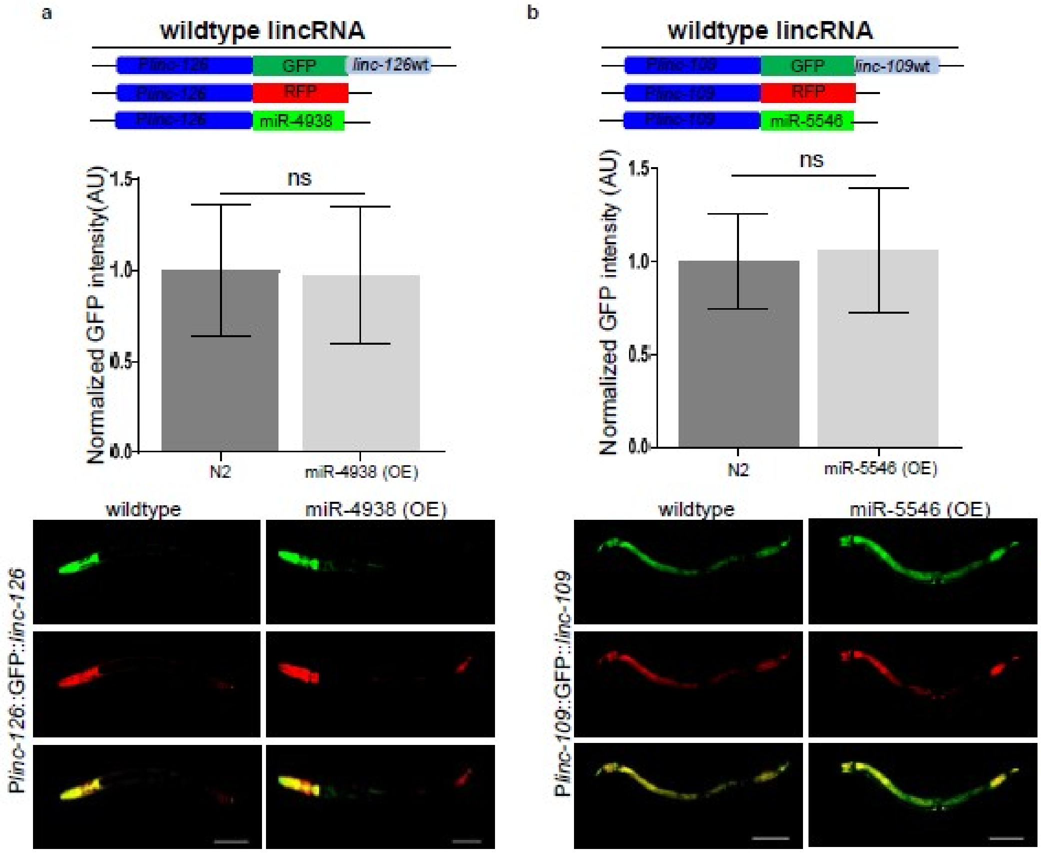
a
wildtype lincRNA
Plinc-126
GFP
linc-126wt
RFP
miR-4938
Normalized GFP intensity(AU)
1.5
1.0
0.5
0.0
ns
N2
miR-4938 (OE)
wildtype
miR-4938 (OE)
Plinc-126::GFP::linc-126
b
wildtype lincRNA
Plinc-109
GFP
linc-109wt
RFP
miR-5546
Normalized GFP intensity (AU)
1.5
1.0
0.5
0.0
ns
N2
miR-5546 (OE)
wildtype
miR-5546 (OE)
Plinc-109::GFP::linc-109
