# Supplementary material for: Systematic evaluation of C. elegans lincRNAs with CRISPR knockout mutants
Source: Genome Biol. 2019 Jan 8;20:7. doi: 10.1186/s13059-018-1619-6 (PMC6325887; doi:10.1186/s13059-018-1619-6)
Supplement: Supplementary file 6 [file 13059_2018_1619_MOESM9_ESM.docx]

Figure S4

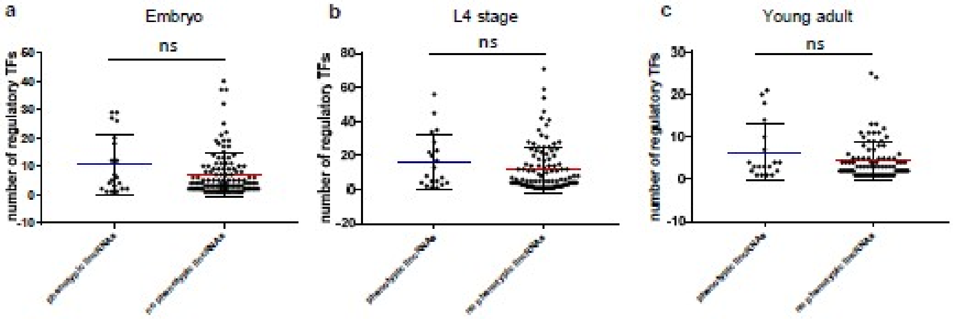
a
Embryo
ns
number of regulatory TFs
60
40
30
20
10
0
-10
b
L4 stage
ns
number of regulatory TFs
80
60
40
20
0
-20
c
Young adult
ns
number of regulatory TFs
30
20
10
0
-10
